# Large dosage Huanglian (Rhizoma Coptidis) for T2DM: A protocol of systematic review and meta-analysis of randomized clinical trials

**DOI:** 10.1097/MD.0000000000022066

**Published:** 2020-09-18

**Authors:** Lizhen Wang, Xiaoying Huang, Rensong Yue, Hongjing Yang, Xinyue Zhang, Yuan Tian, Ning Ding, Linyue Zhou

**Affiliations:** Hospital of Chengdu University of Traditional Chinese Medicine, Chengdu, Sichuan Province, China.

**Keywords:** Huanglian (Rhizoma Coptidis), large dosage,T2DM, systematic review, protocol

## Abstract

**Introduction:**

Type 2 diabetes mellitus(T2DM) is a widespread attention of the world's major health problems. The international diabetes federation (IDF) has released the “global overview of diabetes (ninth edition)”. By 2019. It can lead to complications and even death. Among them, the use of Rhizoma Coptidis (Huanglian) at large dose has also been proved to be effective in clinical practice. However, due to the lack of evidence, there is no specific method or suggestion, so it is necessary to carry out systematic evaluation on coptis coptis and provide effective evidence for further research.

**Methods and analysis:**

We will search the following electronic databases from their inception to May 2020: Electronic database includes PubMed, Embase, Cochrane Library, Web of Science, China National Knowledge Infrastructure. Primary outcomes:fasting blood glucose and glycosylated haemoglobin (A1c). Secondary outcomes: plasma insulin,blood lipid profile,adverse events,and cost associated with the intervention and hospital visit. Data will be extracted by 2 researchers independently, risk of bias of the meta-analysis will be evaluated based on the Cochrane Handbook for Systematic Reviews of Interventions. All data analysis will be conducted by data statistics software Review Manager V.5.3. and Stata V.12.0.

**Results:**

The results of this study will systematically evaluate the effectiveness and safety of large dose of Huanglian intervention for people with T2DM.

**Conclusion:**

The systematic review of this study will summarize the current published evidence of large dose of Huanglian for the treatment of T2DM, which can further guide the promotion and application of it.

**Ethics and dissemination:**

This study is a systematic review, the outcomes are based on the published evidence, so examination and agreement by the ethics committee are not required in this study. We intend to publish the study results in a journal or conference presentations.

Open Science Framework(OSF)registration number: July 21, 2020. https://osf.io/w7bj6

## 1 Introduction

### 1.1 Description of the condition

Type 2 diabetes is a major health concern worldwide. The international diabetes federation (IDF) has released the “global overview of diabetes (ninth edition)”. By 2019, China ranks first in the number of diabetic patients, with a total population of about 116.4 million.^[1,2]^ The second and third most affected countries are India and the United States. It can lead to complications and even death. An estimated $294.6 billion and $109 billion are spent on diabetes-related health in the United States and China, respectively, and about 834,000 people die of diabetes each year in China.^[3]^

### 1.2 Description of the intervention

Routine treatment of type 2 diabetes includes oral hypoglycemic drugs such as metformin, glp-1 agonists and insulin.^[4]^ The reference dosage of rhizoma coptis recorded in the ‘pharmacopoeia of the People's Republic of China’ is 2 to 5 grams, while the dosage for hypoglycemic purposes is usually more than 15 grams, and the maximum dosage for diabetic ketosis can reach 120 grams.^[5]^ Clinical and experimental studies have shown that large dosage Rhizoma Coptidis (Huanglian) (more than 15 grams) for the treatment of type 2 diabetes has the characteristics of rapid hypoglycemic effect, quick effect and stable effect, but the mechanism of action is still unclear.^[6]^

### 1.3 How the intervention might work

Huanglian is a common clinical medicine in traditional Chinese medicine, taken internally to cure thirst (TCM name, symptoms similar to type 2 diabetes). Modern pharmacological research has found that rhizoma coptidis has the functions of anti-microbial, antigenic insect, anti-inflammation, anti-ulcer, anti-cancer, increasing coronary artery blood flow, lowering blood glucose, lowering blood pressure. In recent years, the interaction between intestinal flora and host metabolism has attracted extensive attention. Studies have shown that T2DM can be treated by suppressing the inflammatory response. Large dosage Huanglian can improve the intestinal flora, increase the content of its metabolites short-chain fatty acids, repair the intestinal barrier, and inhibit the activities of histone deacetylase and NF-κB, thus relieving lipolyglycolysaccharide induced inflammation. In addition, they can also participate in the innate immune response and adaptive immune response, playing an important role in intestinal mucosal immune regulation.

### 1.4 Why it is important to this review

The treatment of type 2 diabetes with large dosage Huanglian is a common treatment in traditional Chinese medicine, which is more effective than conventional treatment alone.However, there is no critical evidence for systematic evaluation or meta-analysis of the potential benefits and harms of coptis in the treatment of type 2 diabetes. If this study proves that large dose of Huanglian are truly effective and safe, it will be much easier to use and promote worldwide, and thus benefit more people.

### 1.5 Objectives

The efficacy and harm of large dosage Huanglian in the treatment of type 2 diabetes were systematically evaluated in a randomized controlled trial (RCT). We look forward to providing reference for the treatment of type 2 diabetes in the field of traditional Chinese medicine.

## 2 Methods

### 2.1 Study registration

The protocol of the systematic review has been registered.

Registration: OSF Preregisration. 2020, July 21. https://osf.io/w7bj6 This systematic review protocol will be conducted and reported strictly according to Preferred Reporting Items for Systematic Reviews and Meta-Analyses (PRISMA) ^[7]^ statement guidelines, and the important protocol amendments will be documented in the full review.

### 2.2 Criteria for considering studies for this review

We will strictly screen studies that meet the following inclusion criteria.

#### 2.2.1 Type of included studies

Only RCTs (except Quasi-RCTs and cluster RCTs) will be included. Animal mechanism studies and non-randomised clinical trials will be excluded. Article that substantially overlaps with another published article in print or electronic media will be excluded. Duplicate publications produced by a single experiment and published as separate papers with different criteria for measuring results, priority will be given to original publications and other publications will be excluded. The language and time of publication will not be restricted.

#### 2.2.2 Participants

We will include RCTs of participants of 18 years or older, of any sex, race/ethnicity, and diagnosed with T2DM (diagnosis as defined by the individual trial). We will accept RCTs in which participants had any duration and severity of the disease and were treated with any anti-diabetic therapy. We will exclude trials of patients with type 1 diabetes mellitus or gestational diabetes because of different disease pathways and mechanisms.

#### 2.2.3 Interventions and controls

The interventions included large dosage Huanglian alone or in combination with other conventional treatments. The control group received only conventional treatment. The choice of conventional treatment for each RCT may not be entirely consistent, but large dosage Huanglian alone or in combination with conventional treatment should be the only difference between intervention and control.

#### 2.2.4 Type of outcome measures

Primary outcomes: Mean change in fasting blood glucose (mg/dL) from the baseline; Mean change in glycosylated hemoglobin (%) from the baseline.

Secondary outcomes: Mean change in plasma insulin (μU/ml) from the baseline; Mean change in triglyceride, cholesterol, low-density lipoprotein (LDL), and high-density lipoprotein (HDL) (mg/dL) from the baseline.

Adverse outcomes: Proportion of participants experienced largr dosage Huanglian related adverse events such as abdominal cramping, abdominal pain, nausea, taste disturbance, soft stools, diarrhea, flatulence, bloating, and systemic infection such as septicemia and endocarditis.

Health services outcomes: Costs associated with the intervention; Mean number of hospital or health professional visits.

### 2.3 2.3Search methods

#### 2.3.1 Search resources

**Figure 1:**
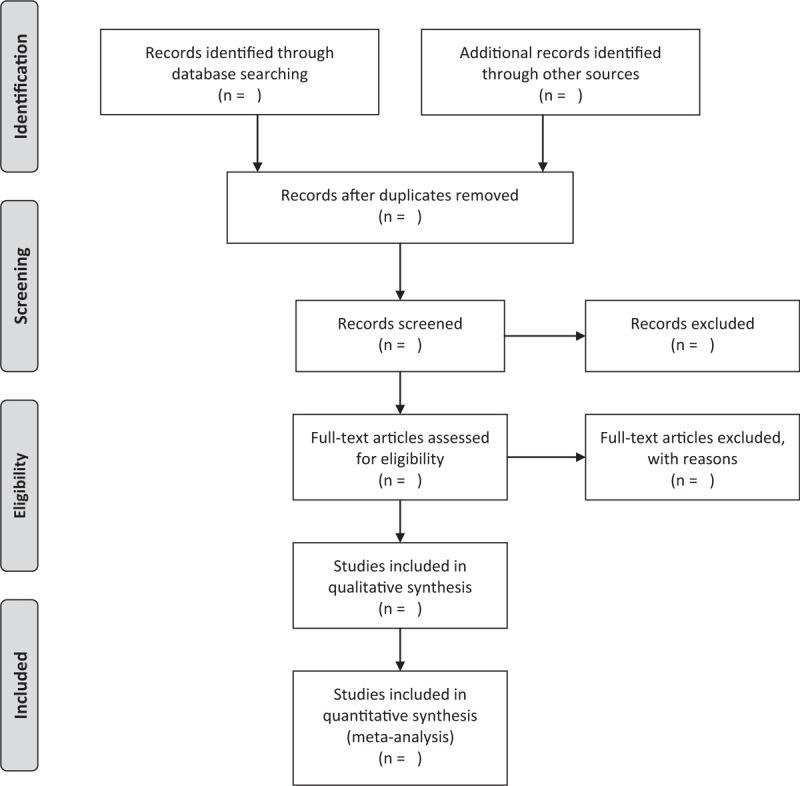
The research flowchart. This figure shows the identification, screening, eligibility and included when we searching articles.

This review will include the following electronic databases from their inception to May 2020: Electronic database includes PubMed, Embase, Cochrane Library, Web of Science, China National Knowledge Infrastructure. (Fig. 1.) The research flowchart.

#### 2.3.2 Search strategies

The following MeSH terms and their combinations will be searched:

(1) Huanglian OR Rhizoma Coptidis;
(2) large dose OR large dosage OR megadose OR more than 15 g;
(3) RCT OR RCTs;
(4) T2DM OR Type 2 diabetes mellitus.

### 2.4 Data collection and analysis

#### 2.4.1 Studies selection

There will be 2 researchers (LW and XH) carry out the selection of research literature independently using endnote x9 software. We will first make the preliminary selection by screening titles and abstracts. Secondly, we will download full text of the relevant studies for further selection according to the inclusion criteria. If there is any different opinion, 2 researchers will discuss and reach an agreement. If a consensus could not be reached, there will be a third researcher (RY) who make the final decision. The details of selection process will be displayed in the PRISMA flow chart.

#### 2.4.2 Data extraction

Two researchers (YT and LZ) will read all the included text in full, and independently extract the following information:

(1) general information, including trial name and registration information;
(2) trial characteristic, in- cluding trial design, location, setting, and inclusion/ex- clusion criteria;
(3) characteristic of participants, including age, sex, race/ethnicity, severity of the diabetes, and comorbidities;
(4) details of interventions, including type, strain, composition of probiotics, dose, duration of treatment, co-interventions (anti-diabetic standard ther- apy);
(5) details of comparison interventions;
(6) out- comes as described under type of outcome measure section.

If we couldn’t reach an agreement, a third researcher(RY) would make the final decision. One researcher (YT) would contact the corresponding author by telephone or e-mail for more information when the reported data were insufficient or ambiguous.

#### 2.4.3 Assessment of risk of bias

All the included studies will be evaluated based on the guidelines of Cochrane Handbook for Systematic Reviews of Interventions.^[8]^ The quality of each trial will categorized into ‘low’, ‘unclear’, or ‘high’ risk of bias according to the following items: adequacy of generation of the allocation sequence, allocation concealment, blinding of participants and personal, blinding of outcome assessors, incomplete outcome data, selected reporting the results and other sources of bias (such as comparable baseline characteristic, inclusion and exclusion criteria).

#### 2.4.4 Assessment of reporting biases

Reporting biases and small-study effects will be detected by funnel plot and Egger's test if there are 10 more studies included in this Meta- analysis. For Egger test, *P* value of <.10 was considered to indicate the exist of reporting biases and small study effects.

#### 2.4.5 Data analysis

We used Revman 5.3 software provided by the Cochrane collaboration to analyze the data. Binary outcomes will be summarized using risk ratio with 95% confidence interval for relative effect. Continuous outcomes will be summarized by using weighted mean difference with 95% confidence interval. We will use random-effect model (REM) for meta-analysis in this review according to research recommendations.^[9]^

Statistical heterogeneity will be assessed by *X*^2^ and *I*^2^ statistical tests. Where *P* value ≥.1and *I*^2^ ≤50%, there is no obvious statistical heterogeneity among the studies. On the contrary, where *P* value <.1or *I*^2^>50% indicates a considerable heterogeneity. Meta-analysis will be performed when the statistical heterogeneity is acceptable (*P* value ≥.1and *I*^2^ ≤50%), otherwise, subgroup analysis will be applied to explore the influence of potential factors on the outcome measures. We will conduct sensitivity analyses by omitting studies 1 by 1 in order to probe the impact of an individual study. If a meta analysis can not be performed, we will conduct descriptive analysis instead.

#### 2.4.6 Patient and public involvement

This is a meta-analysis study based on previously published data, so patient and public involvement will not be included in this study.

#### 2.4.7 Ethics and dissemination

Ethical approval will not be required as this is a protocol for systematic review and meta-analysis. The findings of this study will be disseminated to a peer-reviewed journal and presented at a relevant conference.

#### 2.4.8 Evidence assessed

The quality of evidence for this study will be assessed by “Grades of Recommendations Assessment, Development and Evaluation(GRADE) standard established by the World Health Organization and international organizations.^[10]^ To achieve transparency and simplification, the quality of evidence is divided into 4 levels in GRADE system: high, medium, low and very low. We will employ GRADE profiler 3.2 for analysis.^[11]^

## 3 Discussion

Currently, the effectiveness of coptis in the treatment of T2DM has been confirmed. More and more studies have shown that Huanglian can improve insulin resistance and dyslipidemia,^[12]^ improve chronic metabolic inflammation, improve the damaged intestinal flora, increase the beneficial flora to inhibit the harmful flora, and treat a variety of metabolic diseases including obesity and T2DM. On the other hand, the compound Chinese medicine prescription containing coptis can significantly improve the main symptoms and serum biochemical indicators of T2DM, and significantly improve the quality of life of severe patients with poor conventional treatment.

According to the pharmacological mechanism of rhizoma coptidis, it is believed that its therapeutic effect on T2DM may be related to the alkaloid of rhizoma coptidis. The alkaloids isolated from rhizoma coptidis now include berberine, rhizoma coptidis, epiberberine, bamatine, caprodrine, rhizoma corydalis, rhizoma corydalis, methyl-coptidis, magnolia corydalis, etc. Berberine with antioxidant and anti-inflammatory effect of 2, activating brown adipose tissue (brown adipose tissue) or stimulate white adipose tissue to brown fat cells to promote energy dissipation effect, improve glucose metabolism and insulin resistance and improving islet beta cells secrete insulin 6, its key molecular targets MAPKs, AMPK, the Nrf - 2, the nf-kappa B, glp-1, GLUT, etc. In addition, rhizoma coptidis polysaccharides can significantly inhibit the formation of advanced glycosylated end products, thus alleviating the progression of diabetes and its complications.However, studies have also shown that berberine is the most important toxic component of rhizoma coptidis alkaloids. However, clinical and animal experiments have not yet clarified its mortality and morbidity, and its toxicological mechanism and further studies are needed.Clinical experience has confirmed that compound rhizoma coptidis containing rhizoma coptidis alkaloids is more effective and safer than rhizoma coptidis alkaloids in the treatment of diseases, which also suggests the necessity of considering Chinese medicinal materials instead of chemical extracts in clinical use.

In summary, this systematic review and meta-analysis can help to determine the potential value of coptis in treating T2DM and improving quality of life in critically ill patients. This study can not only provide the basis for the release of diabetes treatment guidelines, but also promote the application of traditional Chinese medicine prescriptions, so as to make more patients benefit.

## Author contributions

**Conceptualization:** Lizhen Wang, Xiaoying Huang, Rensong Yue.

**Data curation:** Yuan Tian, Linyue Zhou, Ning Ding.

**Formal analysis:** Lizhen Wang, Xiaoying Huang.

**Methodology:** Lizhen Wang, Xiaoying Huang, Rensong Yue.

**Project administration:** Rensong Yue.

**Resources:** Yuan Tian, Xinyue Zhang, Hongjing Yang.

**Software:** Lizhen Wang, Xiaoying Huang.

**Supervision:** Rensong Yue.

**Writing – original draft:** Lizhen Wang

**Writing – review & editing:** Rensong Yue.

## Abbreviations

Abbreviations: Huanglian = Rhizoma Coptidis, RCT = randomized controlled trial.
